# Correlation Analysis of Synchronization Type and Degree in Respiratory Neural Network

**DOI:** 10.1155/2021/4475184

**Published:** 2021-12-27

**Authors:** Jieqiong Xu, Quan Yuan, Huiying Chen

**Affiliations:** ^1^School of Mathematics and Information Science, Guangxi University, Nanning 53004, China; ^2^Scientific Research Center of Engineering Mechanics, Guangxi University, Nanning 53004, China; ^3^School of Civil and Architectural Engineering, Guangxi University, Nanning, China

## Abstract

Pre-Bötzinger complex (PBC) is a necessary condition for the generation of respiratory rhythm. Due to the existence of synaptic gaps, delay plays a key role in the synchronous operation of coupled neurons. In this study, the relationship between synchronization and correlation degree is established for the first time by using ISI bifurcation and correlation coefficient, and the relationship between synchronization and correlation degree is discussed under the conditions of no delay, symmetric delay, and asymmetric delay. The results show that the phase synchronization of two coupling PBCs is closely related to the weak correlation, that is, the weak phase synchronization may occur under the condition of incomplete synchronization. Moreover, the time delay and coupling strength are controlled in the modified PBC network model, which not only reveals the law of PBC firing transition but also reveals the complex synchronization behavior in the coupled chaotic neurons. Especially, when the two coupled neurons are nonidentical, the complete synchronization will disappear. These results fully reveal the dynamic behavior of the PBC neural system, which is helpful to explore the signal transmission and coding of PBC neurons and provide theoretical value for further understanding respiratory rhythm.

## 1. Introduction

Synchronous in the neuronal network, a complex population-firing pattern, is believed to play a critical role in many brain functions and many fundamental biological functions. For example, it has been implicated in sensory processing [1], the generation of sleep rhythms [2], the generation of respiratory rhythms [3, 4], Parkinsonian tremor [5], epileptic seizures [6–8], and motor activity [9]. Synchronous arise through interactions between three network components. These are the intrinsic properties of neurons within the network, the synaptic properties of connections between neurons, and the topology of network connectivity. Neurons with intrinsic properties can be described by a large number of mathematical models. One kind of the well-known and biologically plausible models are the Hodgkin–Huxley equations and their simplified versions, which are widely used to analyze the synchronous in the neuronal network [10–12]. When a large number of conducted neuron models are considered, the number of coupled differential equations can be often a problem for computer simulations. Therefore, some models that are simpler but keep some of the dynamical features are considered, such as a discrete-time two-dimensional map proposed by Rulkov, and the integrate-and-fire neural models or hybrid neuron models [13, 14]. Protachevicz et al. investigated how the excitatory and inhibitory connectivities from one brain area to another influence the phase angle and neuronal synchronization, in which the neuron dynamics is given by the adaptive exponential integrate-and-fire model [15]. Borges et al. also used this model to study how spiking or bursting synchronous behavior appears as a function of the coupling strength and the probability of connections [16]. Reis et al. investigated the synchronization properties of a neuronal synchronization model inspired by the connection architecture of the human cerebral cortex using the Rulkov two-dimensional discrete-time map [17]. Recently, functional neuron models were used to connect and couple neural circuits to form a reliable neural network, which could be applied to predict and regulate the collective behaviors of neural networks and neural circuits [18–20]. For example, the synchronization approach between thermosensitive neuron and light-dependent neuron are helpful to detect the changes in environment and heat source exactly [21]. And two piezoelectric sensing neurons (PSNs) were driven by the same external voice for detecting possible synchronization approach without any synapse coupling, which found that two identical PSNs driven by the same periodical stimuli (external forces) could reach synchronous bursting, spiking, and periodical firings, and the synchronization stability was dependent on the external forcing applied on the two PSNs in case of chaotic firing [22]. Except for functional neuron models, field coupling was also considered in the neural circuits recently. Yin et al. used Josephson junction to build a coupling channel for connecting two FitzHugh–Nagumo neural circuits; the hybrid synapse could estimate the effect of the external magnetic field by generating additive phase error between the junction [23]. Yao et al. coupled two Chua circuits via an induction coil coupling, which could benefit the realization of synchronization between two chaotic Chua systems [24].

In the early nineteenth century, a focus of what we now call neuroscience was to discover the noeud vital, that is, the site where the rhythm of breathing originates; 200 years later, the pre-Bötzinger complex (PBC) in the medulla was believed to be the kernel for breathing [25]. Network synchronized activity in the PBC in the mammalian respiratory brainstem controls the inspiratory phase of the respiratory rhythm [26]. To explore the primary kernel for respiratory rhythm in the PBC, Butera et al. presented two minimal models of the PBC and investigated the control and synchronization of coupled neurons [27, 28]. Toporikova and Butera presented a two-compartment mathematical model (TB model) of isolated neurons with two independent bursting mechanisms [29]. Park and Rubin modified the TB model and proposed a single-compartment model (still known as the TB model) [30]. In order to analyze the synchronized activity in a coupled network, we simplify the TB model by considering a parameterized path in a plane, which is of the form of an ellipse with the principal axis along the intracellular calcium concentration axis and the fraction of IP3 (inositol triphosphate) channels axis, to obtain a modified TB model.

A subset of cells within the PBC are able to engage in rhythmic bursting when parameters are adjusted appropriately [31]. Using the methods of dynamical theory, the complex bursting dynamics and their transition mechanisms in the PBC neuron model have been studied widely [32–36]. Recently, Wang et al. considered a model of an isolated embryonic PBC neuron featuring two distinct bursting mechanisms and uncovered mechanisms underlying several different types of intrinsic bursting dynamics observed experimentally including several forms of plateau bursts, bursts involving depolarization block, and various combinations of these patterns [37]. Lü et al. found mixed bursting (MB) could also be driven by the sole action of intracellular calcium oscillations originating from the dendrite based on Park and Rubin's model for a single PBC neuron, which MB was called the dendritic mixed bursting (DMB) [38].

A single neuron exhibits complex nonlinear dynamics behavior, while a group of neurons can show more complicated patterns. In order to understand the dynamics of the neural network, the effects of various forms of coupling connection [39–41] and the time delay [42, 43] are also researched extensively. Synchronization of inspiratory neurons in PBC has an important effect on respiratory rhythm [44]. Gaiteri and Rubin investigated how the dynamics of individual neurons (quiescent/bursting/tonic) and the betweenness centrality of neurons' positions within the network connectivity graph interact to govern network burst synchrony, by simulating heterogeneous networks of PBC neurons [45]. Ashhad and Feldman showed that the inspiratory rhythm emerges as the network reorganizes from random tonic activity toward periodic short-term synchronization [46]. Duan et al. studied the bursting dynamics of the two-coupled PBC complex neurons and explored the possible forms of dynamics that the model network could produce as well the transitions of in- and anti-phase bursting, respectively [47]. They also observed a new type of mixed burst similar to a depolarization block bursting (DB-bursting) in the model of PBC neurons and studied the types of mixed burst and their transition mechanisms by using the multi-time-scale dynamics and one- and two-parameter bifurcation analysis, as well as investigated the effects of persistent sodium conductance on the anti-phase synchronization pattern in coupled PBC neurons [48]. In this paper, using a modified TB model, the relationship between synchronization and correlation degree is established by using ISI bifurcation and correlation coefficient based on the analysis of synchronization and synchronous transition.

The structure of this paper is as follows.  Section 2 first introduces the modified single atrioventricular neuron model under the action of stimulation current, and then the firing interval of this neuron model is determined by using the interspike interval (ISI) bifurcation and phase plane analysis, and the chaos phenomenon in this interval is judged by combining the maximum Lyapunov exponent. At the same time, it is also suspected that the addition period of ISI sequence is related to special bifurcation, which proves that the transition from period 2 to period 3 corresponds to Hopf bifurcation. In Section 3, coupling PBC neural network is considered, and synchronization transition caused by stimulus current, coupling strength, and delay parameters is discussed in three cases: identical and no delay coupling, symmetric time-delay coupling, and asymmetric time-delay coupling. A large number of two-parameter coloring graphs are calculated, which more vividly summarizes the change rule of the synchronization process and proves the continuous dependence of delay equation on coupling strength under weak time delay. In Section 4, the most complex asymmetric nonidentical coupling is considered. In this case, it is difficult for two coupled neurons to achieve complete synchronization. This section mainly analyzes the weak phase synchronization and calculates the similarity function of the membrane potential of two coupled neurons by software simulation (see Appendix for simulation environment). Finally, in Section 5, the relevant conclusions of this paper are summarized.

## 2. Single Compartment PBC Neuron Model

In 2011, Toporikova and Butera [29] established a two-compartment PBC neuron model based on experiments, and then Park and Rubin [30] modified the model to obtain a single-compartment PBC model (still known as the TB model). In this paper, an improved TB model is adopted, which is composed of two parts: the somatic subsystem and the calcium subsystem. The somatic subsystem is affected by calcium-activated nonspecific cationic (CAN) current, while the calcium subsystem is independent of the former. In this paper, we consider a parameterized path in the plane ([Ca], *l*), which is of the form of an ellipse with the principal axis along the [Ca] axis and l axis (see also [49] for similar ideas). The variation along the ellipse can also be regarded as the solution of the equation, as follows:

(1) $$\begin{array}{c} C_{m}\dot{V}=-I_{\text{Na}}-I_{\text{NaP}}-I_{\text{CAN}}-I_{K}-I_{L}-I_{\text{exc}}, \end{array}$$

(2) $$\begin{array}{c} \dot{n}=\frac{\left(n_{\infty }\left(V\right)-n\right)}{\tau_{n}\left(V\right)}, \end{array}$$

(3) $$\begin{array}{c} \dot{h}=\frac{\left(h_{\infty }\left(V\right)-h\right)}{\tau_{h}\left(V\right)}, \end{array}$$

(4) $$\begin{array}{c} \dot{\left[Ca\right]}=-\varepsilon \cdot d\cdot \left(l-l_{c}\right), \end{array}$$

(5) $$\begin{array}{c} \dot{l}=\varepsilon \cdot \frac{1}{d}\cdot \left(\left[\text{Ca}\right]-{\left[\text{Ca}\right]}_{c}\right), \end{array}$$

where [Ca] is the intracellular calcium concentration, *l* is the fraction of IP3(inositol triphosphate) channels in the endoplasmic reticulum membrane that have not been inactivated, [Ca]_*c*_ and *l*_*c*_ define the center of the ellipse, *d* is its aspect ratio, and *ε* is the speed with which the ellipse is traced. See Appendix for the other expression and parameter description of the function in the formula.

**Figure 1:**
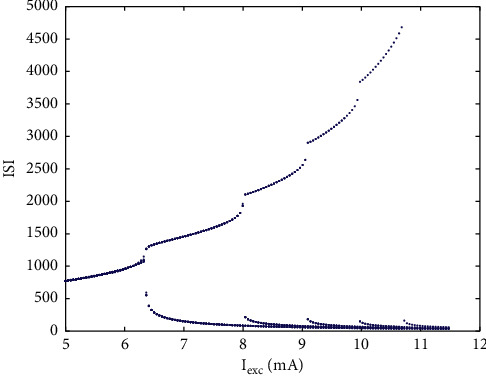
ISI bifurcation diagram of single neuron with parameter *I*_exc_.

**Figure 2:**
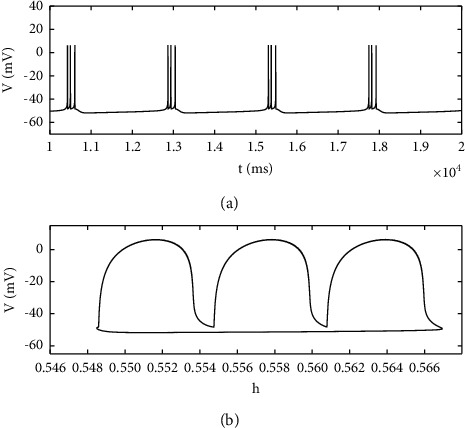
(a) Time history diagram of membrane potential when *I*_exc_=8.5. (b) The phase diagram corresponding to the plane (*h*, *V*).

The stimulation current *I*_exc_ is an important parameter that causes PBC neurons to produce complex firing behavior.  Figure 1 is the ISI bifurcation diagram of *I*_exc_. With the increase of parameters, the number of peaks in the cluster gradually increases, and the ISI sequence of neurons changes from period 1 to period 2 and then increases one by one. However, when the stimulation current increases to 11.5, the ISI sequence disappears, which means that neurons enter a resting state. As shown in Figure 2(a), *I*_exc_=8.5, the time history diagram shows the period 3 bursting, and the phase diagram corresponding to the plane (*h*, *V*) is shown in Figure 2(b).

**Figure 3:**
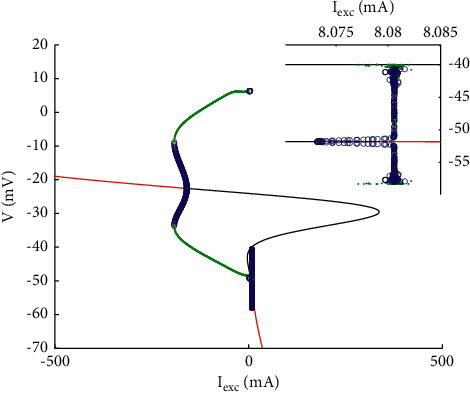
Bifurcation diagram of a single PBC neuron with respect to parameter *I*_exc_. The upper right corner shows the local enlarged view.

It is worth noting that the threshold value of period-adding bifurcation may correspond to a special bifurcation type, and the relationship between them is discussed from the perspective of bifurcation. The bifurcation diagram of the system with respect to parameter *I*_exc_ is shown in Figure 3. The solid line is the bifurcation curve of the equilibrium point, and its trend is classical cubic, in which red and black represent stable and unstable equilibrium points, respectively. The lower branch of the curve consists of a stable node and an unstable saddle point, which changes through Hopf bifurcation point (HB_2_); then turns to the middle branch via first fold bifurcation (LF), which is an unstable equilibrium point; and finally turns to the upper branch of the equilibrium point curve through the second fold bifurcation (RF), which is basically the same as the lower branch. With the decrease of parameters, it changes from unstable to stable, and the limit cycle bifurcation occurs at HB_1_, in which green and blue represent stable and unstable periodic orbits, respectively, and the limit cycle changes from unstable to stable through saddle node on the invariant cycle (SNIC) and finally disappears into the equilibrium bifurcation curve, and the end point is homoclinic bifurcation (HC). According to this idea, the limit cycle bifurcation can also be extended in HB_2_(*I*_exc_=8.073), and the enlarged figure is shown in the upper right corner. Although the structure type is basically the same as that at HB_1_, it is a small change, and there are pitchfork bifurcation (BP, *I*_*exc*_=8.0805) and period doubling bifurcation (PD, *I*_exc_=8.08193), which is similar to the period in Figure 1. It can be judged that the change of neuron peak has a great relationship with the type of bifurcation point, especially HB_2_. Now calculate the first Lyapunov coefficient at this point to judge the direction of Hopf bifurcation as follows:

(6) $$\begin{array}{c} A={\left(\begin{array}{ccc} \frac{\partial f_{1}}{\partial V} & \frac{\partial f_{1}}{\partial n} & \frac{\partial f_{1}}{\partial h}\\ \frac{\partial f_{2}}{\partial V} & \frac{\partial f_{2}}{\partial n} & \frac{\partial f_{2}}{\partial h}\\ \frac{\partial f_{3}}{\partial V} & \frac{\partial f_{3}}{\partial n} & \frac{\partial f_{3}}{\partial h} \end{array}\right)|}_{x_{0}}=\left(\begin{array}{ccc} 0.00010974 & -0.00283353 & 1.18619\\ 0.000718508 & -0.869859 & 0\\ -0.00000465547 & 0 & -0.000107 \end{array}\right)\left(\text{L}1\right), \end{array}$$

(7) $$\begin{array}{c} B\left(x,y\right)=\left(\begin{array}{c} 0.0135x_{1}y_{1}-0.0016x_{1}y_{2}-0.0016x_{2}y_{1}+0.1619x_{1}y_{3}+0.1619x_{3}y_{1}-0.0023x_{2}y_{2}\\ 0.1080x_{2}y_{2}+0.1080x_{1}y_{1}\\ 0.00005\cdot \left(0.3918x_{3}y_{1}+0.3918x_{1}y_{3}\right) \end{array}\right)\left(\text{L}2\right), \end{array}$$

(8) $$\begin{array}{c} C\left(x,y\right)=\left(\begin{array}{c} x_{1}\left(0.0012y_{1}z_{1}-0.0009y_{1}z_{2}-0.0009y_{2}z_{1}+0.0184y_{1}z_{3}+0.0184y_{3}z_{1}-0.0001y_{2}z_{2}\right)\\ +x_{2}\left(-0.0009y_{1}z_{1}-0.0001y_{1}z_{2}-0.0001y_{2}z_{2}-1.4077y_{2}z_{2}\right)+x_{3}\left(0.0184y_{1}z_{1}\right)\\ x_{1}\left(-0.0136y_{1}z_{2}-0.0136y_{2}z_{1}\right)+x_{2}\left(-0.0136y_{1}z_{1}\right)\\ l1x\left(-0.0047y_{1}z_{1}-0.1074y_{1}z_{3}-0.1074y_{3}z_{1}\right)+0.00005x_{3}\left(-0.1074y_{1}z_{1}\right) \end{array}\right)\left(\text{L}3\right). \end{array}$$

The equilibrium point of the original system at HB_2_ is *x*_HB_=(−51.8239, 0.003315, 0.6824, 0.1, 0.9), but because the calcium subsystem is independent, we only need to fix [Ca] at the equilibrium ([Ca]=0.1) and discuss the somatic subsystem. The Jacobian matrix at the point is (6), and the expression of function *F*=(*f*_1_, *f*_2_, *f*_3_)^*T*^ is shown in Appendix. The matrix has a pair of conjugate complex roots *λ*_1,2_=±*ωi*, *ω*=0.0023475; the other eigenvalue is *λ*=−0.869856; and the eigenvector corresponding to the positive imaginary root is

(9) $$\begin{array}{c} q={\left(1,0.000826-2.2291e-6i,-0.000090541+0.001979i\right)}^{T}. \end{array}$$

Take another vector *p* satisfying *A*^*T*^*p*=−*ωip* and 〈*p*, *q*〉=1 and get

(10) $$\begin{array}{c} p={\left(0.000090542-0.001979i,-3.1233e-7+6.4457e-6i,1\right)}^{T}. \end{array}$$

Consider system $\dot{x}=Ax+F\left(x\right),x\in R^{n}$. *A* is the Jacobian matrix at the equilibrium point, which can be written as follows:

(11) $$\begin{array}{c} F\left(x\right)=\frac{1}{2}B\left(x,x\right)+\frac{1}{6}C\left(x,x,x\right)+O\left({\left\|x\right\|}^{4}\right), \end{array}$$

where *B*(*x*, *y*) and *C*(*x*, *y*, *z*) are multiple-linear functions with component form, and the expression is

(12) $$\begin{array}{c} B_{i}\left(x,y\right)={\sum_{j,k=1}^{3}\frac{\partial^{2}F_{i}\left(\xi \right)}{\partial \xi_{j}\partial \xi_{k}}|}_{\xi =0}x_{j}y_{k},\;i=1,2,3,\\ C_{i}\left(x,y,z\right)={\sum_{j,k,l=1}^{n}\frac{\partial^{3}F_{i}\left(\xi \right)}{\partial \xi_{j}\partial \xi_{k}\partial \xi_{l}}|}_{\xi =0}x_{j}y_{k}z_{l},\;i=1,2,3, \end{array}$$

where *ξ*=(*ξ*_1_, *ξ*_2_, *ξ*_3_) is the equilibrium of the system, which can be obtained from equations (7) and (8).

It can be calculated accordingly:

(13) $$\begin{array}{c} g_{20}=\langle p,B\left(q,q\right)\rangle =3.4149e-08+2.6785e-05i,\\ g_{11}=\langle p,B\left(q,\bar{q}\right)\rangle =1.4659e-05+3.2040e-04i,\\ g_{21}=\langle p,C\left(q,q,\bar{q}\right)\rangle =-0.0047. \end{array}$$

The first Lyapunov coefficient is

(14) l10=12ω2Reig20g11+ωg21=−1.0025.

Therefore, the direction of Hopf bifurcation is supercritical. The detailed derivation of the first Lyapunov coefficient can be referred to reference [50].

## 3. Chaotic Coupled PBC Model

The dynamic behavior of two electrically coupled (linearly coupled) PBC neurons consists of eight differential equations including Eqs. (4) and (5) and the following equations:

(15) $$\begin{array}{c} C_{m}\dot{V_{i}}=-I_{Na_{i}}-I_{NaP_{i}}-I_{{\text{CAN}}_{i}}-I_{K_{i}}-I_{L_{i}}-I_{{\text{exc}}_{i}}-I_{c_{i}}, \end{array}$$

(16) $$\begin{array}{c} \dot{n_{i}}=\frac{\left(n_{\infty }\left(V_{i}\right)-n_{i}\right)}{\tau_{n}\left(V_{i}\right)}, \end{array}$$

(17) $$\begin{array}{c} \dot{h_{i}}=\frac{\left(h_{\infty }\left(V_{i}\right)-h_{i}\right)}{\tau_{h}\left(V_{i}\right)}, \end{array}$$

where *i*=1,2 stands for neurons 1 and 2. Because the two subsystems have unidirectional influence, they act on the same calcium subsystem (4, 5) when coupling two neurons. This section mainly studies the synchronization behavior of two identically coupled chaotic PBC neurons. By calculating the ISI bifurcation, correlation coefficient, and maximum synchronization difference of the two coupled neurons, it shows the rich synchronization transition modes of PBC neurons. Fixed *I*_exc1_=*I*_exc2_=8.5, the correlation coefficient *R* and the maximum synchronization difference max (*e*) are defined as follows:

(18) $$\begin{array}{c} R=\frac{\sum_{k=1}^{N}\left(V_{1}^{k}-{\bar{V}}_{1}\right)\left(V_{2}^{k}-{\bar{V}}_{2}\right)}{\sqrt{\sum_{k=1}^{N}{\left(V_{1}^{k}-{\bar{V}}_{1}\right)}^{2}\cdot \sum_{k=1}^{N}{\left(V_{2}^{k}-{\bar{V}}_{2}\right)}^{2}}}, \end{array}$$

(19) $$\begin{array}{c} \max\left(e\right)={\left\|V_{1}-V_{2}\right\|}_{\infty }=\underset{1\leq i\leq N}{\max}\left(V_{1}-V_{2}\right), \end{array}$$

where *N* is the total number of samples, *V*_*i*_ is the membrane potential of the neuron *i*, ${\bar{V}}_{i}$ is the corresponding average value, and ‖·‖_*∞*_ is the infinite norm.

### 3.1. Complete and Incomplete Synchronization

In this section, the synchronous transition caused by coupling strength is mainly analyzed when there is no delay coupling, as shown in equation (15).

**Figure 4:**
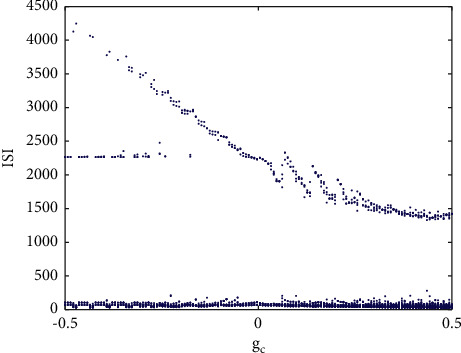
ISI bifurcation diagram of coupling strength *g*_*c*_.

Here, *g*_*c*_ is the coupling strength. When two neurons start to couple, a single neuron will also show different firing patterns, as shown in Figure 4, which shows the relationship between the ISI of the first neuron and the coupling strength *g*_*c*_. When *g*_*c*_ ∈ (−0.5, −0.3), neuron 1 will always period-3 bursting, which is the same as the action potential of neurons in Figure 2. With the increase of coupling strength, the size of the attractor of coupled neurons tends to increase. In this case, the coupling strength of the chaotic system destroys the attractor with three cycles in a single neuron, resulting in more complex attractors. When the two neurons are positively coupled, this situation becomes more obvious, and the peak value in the corresponding bursting becomes more irregular.

In addition, the ISI bifurcation diagram has obvious high and low parts. The higher ISI sequence corresponds to the resting state, and it will gradually decrease with the increase of coupling strength.

**Figure 5:**
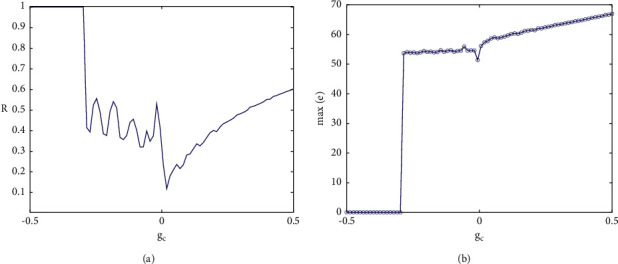
(a) The variation between the correlation coefficient and coupling strength *g*_*c*_ of two coupled neurons. (b) Change diagram between maximum synchronization difference and coupling strength *g*_*c*_.

**Figure 6:**
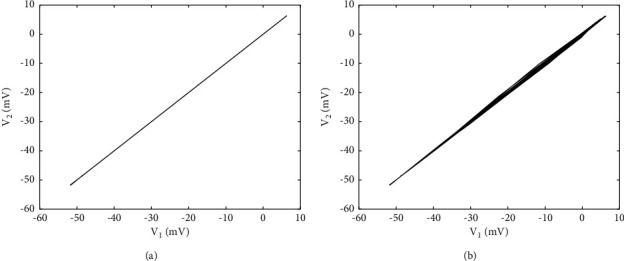
The phase diagram of two coupled PBC neurons on the (*V*_1_, *V*_2_) plane when the coupling strength is different: (a) *g*_*c*_=−0.5 and (b) *g*_*c*_=−0.24.

The coupling strength is not only an important factor affecting the firing patterns of neurons but also a key parameter affecting the synchronous transition of neural networks. It can be observed from Figures 5(a) and 5(b) that when the coupling strength is between (−0.5, −0.3), the correlation coefficient of membrane potential between the two neurons is always 1, and the maximum synchronization difference is always 0, which means that the two coupled neurons are completely synchronized, but with the increase of the coupling strength, they start to become incompletely synchronized. Complete synchronization and incomplete synchronization are two extreme cases, which can be directly judged by a correlation coefficient, while there are other types of synchronization under incomplete synchronization. As shown in Figure 6(a), when *g*_*c*_=−0.5, the phase diagram of the coupled system on the (*V*_1_, *V*_2_) plane coincides with the 45-degree bisector, which again indicates that the coupled neurons are in-phase synchronous state, that is, complete synchronization. With the increase of coupling strength, the degree of synchronization gradually weakens (the correlation coefficient drops sharply in Figure 5(a)). In Figure 6(b), when *g*_*c*_=−0.24, the action potentials of the two coupled neurons are almost identical, but in the phase diagram on the (*V*_1_, *V*_2_) plane, a small disturbance around the 45-degree bisector can be clearly observed, which indicates that the firing sequences of the two neurons are almost coincident but also completely overlapped, so it is called almost in-phase synchronous state.

**Figure 7:**
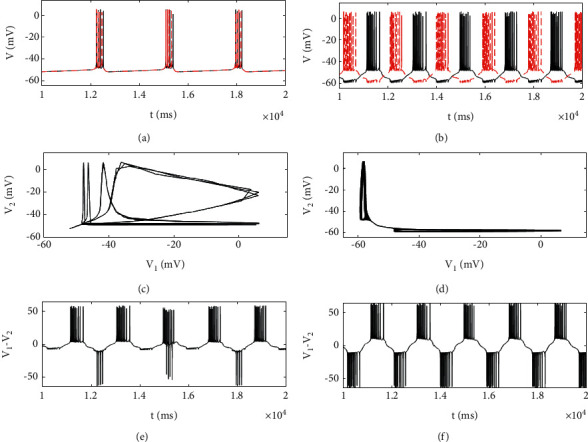
The phase diagrams on different planes with different values of coupling strength, *g*_*c*_=−0.1 in the left column and *g*_*c*_=0.4 in the right column. (a) and (b) Time history diagram of the membrane potential of two neurons: black and red are neurons 1 and 2, respectively. (c) and (d) The phase diagram in planes (*V*_1_, *V*_2_). (e) and (f) Synchronization difference of membrane potential.

In addition, in Figure 5(b), the maximum value of synchronization difference jumps rapidly with the increase of coupling strength to *g*_*c*_=−0.3, which also indicates that complete synchronization is lost and remains basically unchanged in a certain range. When the positive coupling is achieved, the maximum synchronization difference tends to increase monotonously. If the synchronization difference is nonzero, it only means that the two neurons are not fully synchronized, and it is not certain that the coupled neurons are out of synchronization, such as phase synchronization, lag synchronization, and chaotic synchronization, which may occur. As shown in Figure 7(a), the periodic bursting of two coupled neurons occur at the same time, and the neurons generate irregular electricity, and the corresponding phase diagram is in an irregular state, and there is no fixed phase between the two firing sequences, indicating that the coupled neurons are in an asynchronous state (Figure 7(c)).

When *g*_*c*_=0.4, the phase diagram is symmetrical about the 45-degree bisector, and the coupled neurons are out-of-phase synchronization. Both neurons produce almost the same bursting, but the oscillation time of action potential is inconsistent, which is one of the neuron action potentials, another in the resting state, and the time history diagram of poor synchronization also shows periodic phenomenon (Figure 7(f)). In the respiratory system, when the expiratory neurons produce exhalation, the inspiratory neurons are usually resting, and when the inspiratory neurons operate, the expiratory neurons are also in the resting state. In the respiratory system, when the expiratory neurons produce exhalation, the inspiratory neurons are usually resting, and when the inspiratory neurons operate, the expiratory neurons are also in the resting state. This phenomenon has also been found in other biological behaviors, such as the walking of limb animals, the formation of a central pattern generator (CPG), and the alternating arm swinging behavior of runners.

### 3.2. The Continuous Dependence on the Coupling Strength under Weak Delay

In the real biological system, because the information transmission is carried out at a limited speed, the time delay is inevitable in the process of information transmission, which makes the dynamic system exhibit rich dynamic characteristics, such as phase locking, synchronization, and multi-stability, which shows that the time delay is of great significance in information processing based on neural movement.

In equation (15), *I*_*c*_*i*__ satisfies *I*_*c*_*i*__=*g*_*c*_ · (*V*_*j*_^*τ*^ − *V*_*i*_), where *τ* is the time delay, and *i*, *j* ∈ {1,2}, and *i* ≠ *j*.

This section mainly discusses the synchronization behavior caused by time delay. According to the relationship between two coupled neurons without time delay in the case of identical coupling, the synchronization effect of time delay on the coupled system is considered when the coupling strength *g*_*c*_=−0.5 and *g*_*c*_=0.4, respectively. As before, the infinite norm of correlation coefficient and synchronization difference is still used to judge the synchronization between coupled neurons.

**Figure 8:**
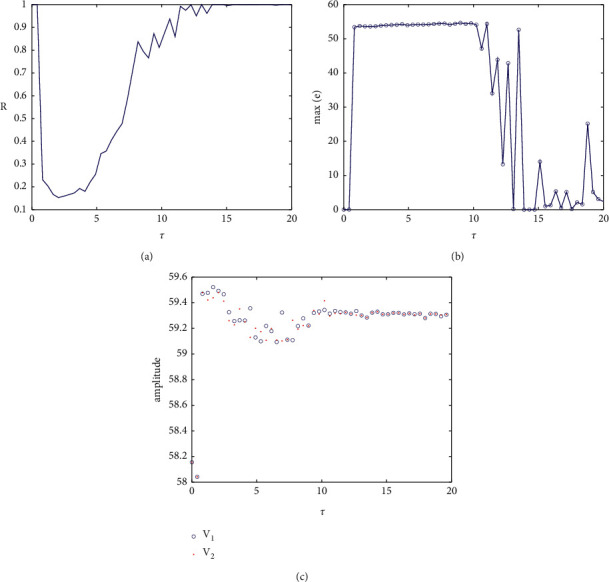
When *g*_*c*_=−0.5: (a) correlation coefficient of membrane potential of coupled neurons with time delay, (b) a graph showing the relationship between the maximum synchronization difference and the change of *τ*, and (c) the relationship between the amplitude of two PBC neurons and *τ*.

When *g*_*c*_=−0.5, according to definitions (18) and (19), the relationship between the correlation coefficient and the maximum synchronization difference with time delay is shown in Figure 8. When the time delay is 0, it is the situation discussed above. The two coupled neurons are completely synchronized and remain in a complete synchronization state with the increase of the small range of time delay. When the delay increases to 10, the maximum synchronization difference is not 0 and basically remains unchanged, and the corresponding correlation coefficient is also very small. When the correlation coefficient is less than 0.3, it can basically be considered that there is no correlation between the two statistics, which can also be used to explain that the two coupled neurons are not completely synchronized at *τ* < 10. Combined with Figure 8(c), the amplitudes of the two neurons in this range are also inconsistent, thus judging that the two neurons are asynchronous. However, when the time delay is large, the correlation coefficient is close to 1, and the corresponding maximum synchronization difference fluctuates in a low amplitude state, which means that the two neurons transform between complete synchronization and approximate synchronization and are infinitely close to complete synchronization. When the *τ* ∈ (10,14), the fluctuation of correlation coefficient increases and the corresponding maximum synchronization difference gradually decreases, indicating that the asynchronous state disappears and the coupled neurons reach the transitional stage of synchronization.

**Figure 9:**
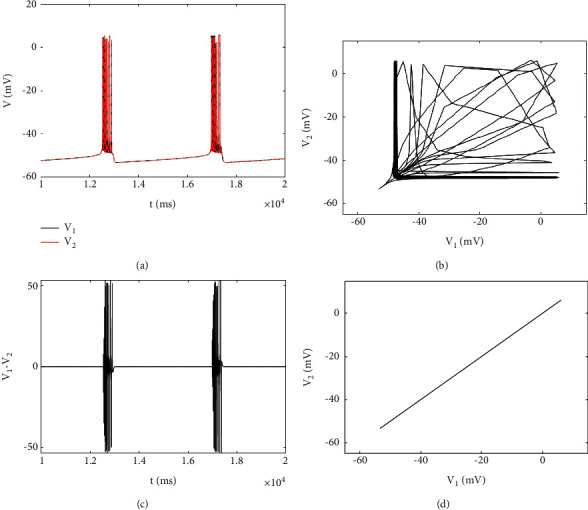
Phase plan diagram of two PBC neurons: (a) the time history diagram of coupled neurons when *τ*=5, (b) the phase diagram in the plane (*V*_1_, *V*_2_) when *τ*=5, (c) time history diagram of synchronization difference when *τ*=5, and (d) phase diagram in plane (*V*_1_, *V*_2_) when *τ*=17.

In order to better observe the effect of the time delay on the synchronization, the release sequences of two neurons are obtained when *τ*_1_=5, as shown in Figure 9(a). The release sequences of two neurons is consistent basically, and resting and active stage of bursting synchronization is achieved. However, through the difference of synchronization in Figure 9(c), it is not difficult to find that in the firing sequence of membrane potential, the firing time of two coupling neurons is different, which indicates that the two neurons are bursting synchronization with unrelated peaks, and the situations in Figures 9(b) and 9(c) are completely consistent, indicating that two coupling neurons remain chaotic when the delay is increased.  Figure 9(d) is the phase diagram of the system on the (*V*_1_, *V*_2_) plane when *τ*=17, which coincides with the 45 degree, which means that the two neurons are completely synchronized.

**Figure 10:**
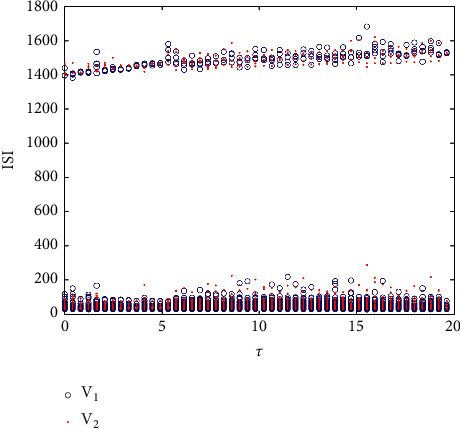
Bifurcation diagram of ISI and *τ* in two coupled neurons when *g*_*c*_=0.4.

**Figure 11:**
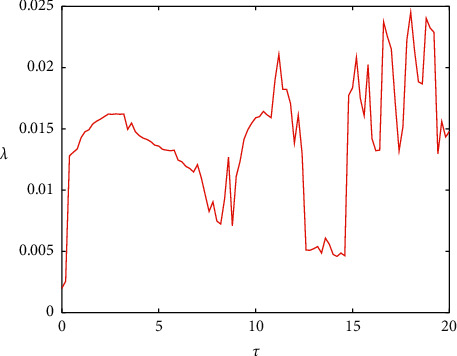
The maximum Lyapunov exponent with time delay *τ*.

**Figure 12:**
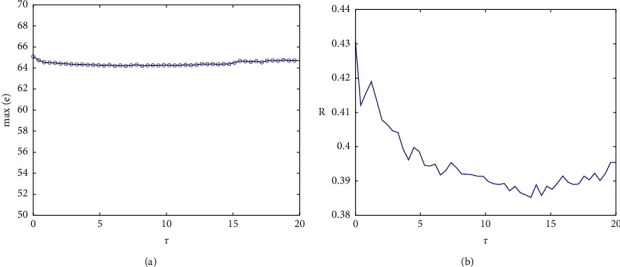
(a) The relationship between the maximum synchronization difference of the membrane potential of coupling neurons and *τ*. (b) The change diagram of correlation coefficient *R* and *τ* when *g*_*c*_=0.4.

When *g*_*c*_=0.4, the ISI sequences of the two neurons are as shown in Figure 10, which consists of two parts. The higher sequence is the resting period, and the resting state increases slightly with the change of time delay. The lower sequence corresponds to the firing state of the action potential, and the peak number in the bursting changes obviously in the middle lag. On the whole, the ISI sequences of the two neurons are deviated, indicating that the membrane potential is not completely synchronized. Figure 10 shows that the system will show chaos under time delay coupling, which is also illustrated by the maximum Lyapunov exponent in Figure 11, but the chaos at this time is completely caused by time delay, instead of the transition from period to chaos in Figure 4. In this complicated situation, the correlation coefficient and the maximum potential difference are still used to judge. As shown in Figure 12, the maximum synchronization difference is kept around 65 mV, and the corresponding correlation coefficient is also small, and the fluctuation decreases with the increase of *τ*. It will be shown that although the two coupling neurons are not completely synchronized in this case, they can achieve weak synchronization.

When discussing identical coupling no delay, the synchronization phenomenon caused by coupling strength *g*_*c*_ has been analyzed. When *g*_*c*_=−0.5, the coupled neurons are completely synchronized, which changes from no delay to symmetric time delay. Whether there is a mutation or continuous dependence on parameters needs further analysis. In Figure 8, it is found that in the case of weak delay (*τ* ∈ (0,0.02)), the coupling neurons have a high degree of synchronization, which can be understood as the continuous dependence of the equation on the parameters. The continuous dependence is mainly for ordinary differential equations, but the time delay system is not fully sure. Now, we analyze the dependence of the system on the coupling strength under weak delay.

First, the coupled system is considered as follows:

(20) $$\begin{array}{c} {\dot{u}}_{i}=f\left(u_{i}\right)+\tilde{C}\left(u_{j}^{1}-u_{i}^{1}\right),\;u_{i}\in R^{n}, \end{array}$$

where $\tilde{C}\left(u_{j}^{1}-u_{i}^{1}\right)$ is the feedback strength, *i*, *j*={1,2}, *i* ≠ *j*, $\tilde{C}=\left(C,\underset{n-1}{\underset{︸}{0,0,\ldots ,0}}\right)$, and *f* is a nonlinear differentiable function. The common method to study the synchronization problem of the coupled system is to deal with the synchronization error, which can be obtained from (20) by making *e*=*u*_2_ − *u*_1_

(21) $$\begin{array}{c} \overset{\cdot }{\;e}=f\left(u_{2}\right)-f\left(u_{1}\right)-2\tilde{C}\left(u_{2}^{1}-u_{1}^{1}\right). \end{array}$$

The system is linearized at *e*=0; we have

(22) $$\begin{array}{c} \dot{e}=\left(Df\left(u\right)-M\right)e, \end{array}$$

where $M={\left(\begin{array}{cccc} 2C & 0 & \cdots & 0\\ 0 & 0 & \cdots & 0\\ \vdots & \vdots & \ddots & \vdots \\ 0 & 0 & \cdots & 0 \end{array}\right)}_{n\times n}$, if *Df*(*u*) is the Jacobian matrix of *f*(*u*); it is assumed that there exists Lyapunov function *L*(*e*), which satisfies (23). The coupling system (20) is said to be synchronized.

(23) $$\begin{array}{c} \forall e\neq 0\Rightarrow L\left(e\right)>0, \end{array}$$

(24) $$\begin{array}{c} \overset{\cdot }{u_{i}}=f\left(u_{i}\right)-C\left(\begin{array}{c} 0\\ 0 \end{array}\right)+C\left(\left(\begin{array}{c} 0\\ 0 \end{array}\right)-\tau \left(f\left(u_{j}\right)-C\left(\begin{array}{c} 0\\ 0 \end{array}\right)\right)\right),\;i,j\in \left\{1,2\right\},i\neq j. \end{array}$$

For the system used in this paper, *τ*_1_=*τ*_2_=*τ* ≠ 0, under weak time delay, *x*(*τ* − *t*) Taylor expansion is obtained as follows:

(25) $$\begin{array}{c} x\left(\tau -t\right)=x\left(t\right)-\tau \overset{\cdot }{x\left(t\right)}+\text{O}\left(\tau^{2}\right). \end{array}$$

Since *τ* is in a small range, the higher-order term in the formula can be ignored and can be obtained equation (24). Hence,

(26) $$\begin{array}{c} \dot{e}=\overset{\cdot }{u_{2}}-\overset{\cdot }{u_{1}}=\left(1+\tau C\right)\left(f\left(u_{2}\right)-f\left(u_{1}\right)\right)-2C\left(1+\tau C\right)\left(\begin{array}{c} e_{1}\\ 0\\ 0 \end{array}\right). \end{array}$$

Linearize at *e*=0 and get

(27) $$\begin{array}{c} \dot{e}=\left(1+\tau C\right)\left(Df\left(u\right)-M\right)e, \end{array}$$

where $\dot{e}=\left(1+\tau C\right)\left(Df\left(u\right)-M\right)e$. From the point of view of dynamics, the stability of the system (20) depends only on *M*, that is, *C*. On the other hand, the synchronization dynamics of a system with weak time delay can be transformed into the stability of the system (20), so the synchronization in this small range can be determined by the coupling strength.

**Figure 13:**
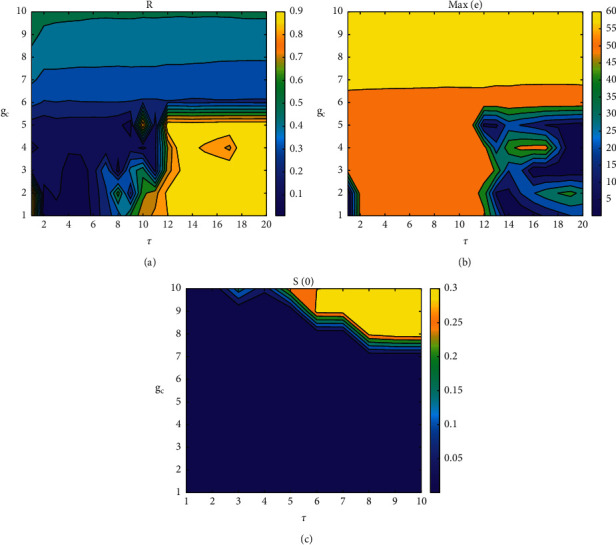
Correlation coefficient and maximum synchronization difference of coupled neuron membrane potential in two-parameter space (*τ*, *g*_*c*_): (a) correlation coefficient, (b) maximum synchronization difference, and (c) similarity functions in the local neighborhood of graphs (a) and (b).

The correlation coefficient and the maximum synchronization difference are observed in the two-parameter space (*τ*, *g*_*c*_) (Figure 13). When *g*_*c*_ > 0, the correlation coefficient is low, and the maximum synchronization difference is too large. At this time, for any time delay, the coupled system will not achieve complete synchronization, which means that the time when the two neurons' membrane potential firing sequence is always inconsistent in the case of positive coupling, and it will not achieve bursting synchronization with the change of *τ*, which is obvious in negative coupling. It is mainly divided into two parts, *τ* < 10 and *τ* > 10. When *τ* > 10, the correlation coefficient is on the high side, and the transition from approximate synchronization to complete synchronization is gradually realized with the influence of coupling strength. When *τ* < 10, the correlation coefficient is low, and the maximum synchronization difference is always nonzero, which indicates that the two coupling neurons are asynchronous. In the local neighborhood of time delay and coupling strength, their similarity functions by defined (30) are mostly 0 (Figure 13(c)), indicating that neurons are completely synchronized. This phenomenon can also be explained as the continuous dependence of the delay equation on parameters, which is completely consistent with the previous derivation.

### 3.3. The Relationship between Phase Synchronization and Weak Correlation under Asymmetric Time-Delay Coupling

For different neurons, the speed of information transmission is also different, so it is necessary to discuss the asymmetric time-delay coupling system. The emergence of asymmetric time delays makes the finite-dimensional system become an infinite-dimensional system, thus inducing more complex dynamic characteristics.

(28) $$\begin{array}{c} I_{c_{i}}=g_{c}\cdot \left(V_{j}^{\tau_{i}}-V_{i}\right), \end{array}$$

where *τ*_1_ ≠ *τ*_2_, *i*, *j* ∈ {1,2}, and *i* ≠ *j*.

**Figure 14:**
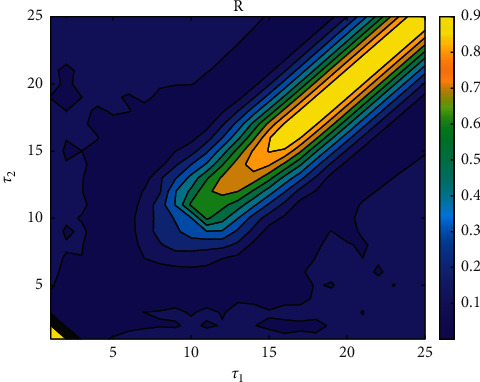
Correlation coefficient of asymmetric time-delay coupled neurons in two-parameter plane when *g*_*c*_=−0.5.

The correlation coefficient of two neurons coupled with asymmetric time delays on the two-parameter plane (*τ*_1_, *τ*_2_) is shown in Figure 14, and the correlation degree of the diagonal part is obviously higher than that of other places, which is caused by the complete synchronization caused by the strong symmetric time delay discussed in the previous section, which again conforms to the continuous dependence of the time-delay equation on parameters mentioned above, and this is applicable not only to weak time delay but also to strong time delay. However, in the nondiagonal region, the correlation coefficient is obviously low, which means that the complete synchronization of the two coupled neurons is lost from symmetric time delay to asymmetric time-delay coupling. When the correlation coefficient *R* = 1, it means that the coupling neurons are completely synchronized. When the correlation coefficient is high but not equal to 1, it indicates that the neurons may be from a certain degree of approximate synchronization to complete synchronization. When the correlation coefficient is high but not equal to 1, it indicates that the neurons may be from a certain degree of approximate synchronization to complete synchronization. The former case of incomplete synchronization is mainly judged by phase plane analysis, which has obvious limitations and cannot explain the characteristics in the general state.

A complete synchronization is a kind of synchronization behavior achieved by two identical chaotic systems, but the interacting chaotic systems usually are not identical in real life, which makes it difficult for coupled chaotic systems to achieve complete synchronization, but other synchronizations cannot be excluded. Therefore, it is necessary to study weak synchronization behavior, and phase synchronization is one of them. In order to study phase synchronization, it is necessary to define the phase of chaotic systems. At present, there are many known methods, and the commonly used method is an analytical signal approximation and Poincaré mapping, there we use the Poincaré mapping definition phase.

(29) $$\begin{array}{c} \phi \left(t\right)=2\pi \frac{t-t_{n}}{t_{n+1}-t_{n}}+2\pi n,t_{n}\leq t\leq t_{n+1}, \end{array}$$

where *t*_*n*_ is the time for the trajectory to cross the Poincaré graph for the *n* time, if the phase difference Δ*ϕ*=|*ϕ*_1_(*t*) − *ϕ*_2_(*t*)| < const, the two coupled neurons are said to be in phase synchronization. This paper limits const ≤ 2*π*.

**Figure 15:**
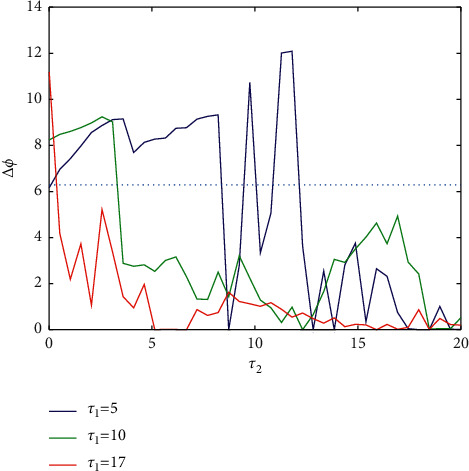
The phase difference between the two coupled neurons and *τ*_2_ when different *τ*_1_.

First, fixed *g*_*c*_=−0.5, as shown in Figure 14, the correlation coefficient in the nondiagonal region shows a symmetrical decreasing trend, and the vertical line *τ*_1_=5 is taken in the figure, and the phase difference about *τ*_2_ is as shown in Figure 15, most of which lies above the baseline (2*π*), mainly corresponding to the part where *R* < 0.1 in Figure 14, which indicates that the coupled neurons are asynchronous, and the diagram corresponding to the phase plane is similar to that in Figures 7 and 9. Jumping near the baseline means that neurons transform between asynchronous and phase synchronization. When the correlation coefficient is greater than 0.2 (i.e., *τ*_2_ > 14), the two coupled neurons are always in phase synchronization.

**Figure 16:**
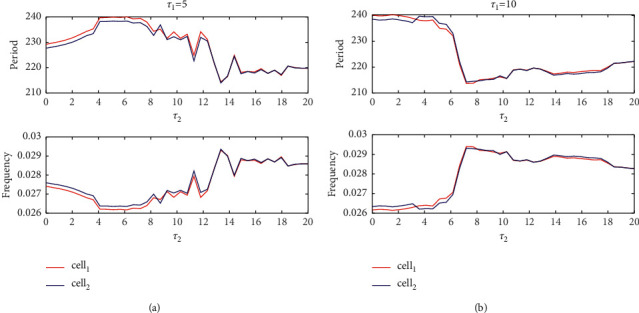
The relationship between period and amplitude with *τ*_2_ when different *τ*_1_.

When *τ*_1_=10, the coupled neurons were still within a certain range of asynchronous, but the scope was obviously smaller than *τ*_1_=5, and then they are always in phase synchronization. When *τ*_1_ increases to 17, this situation is more prominent, and it is asynchronous in a small range of time-delay changes, while the remaining ranges are all phase synchronization. The Poincaré mapping is used to analyze the period and frequency.  Figure 16 shows the period and frequency of different *τ*_1_, and red and blue correspond to cell 1 and cell 2, respectively. Obviously, the period and frequency are inversely proportional. In Figure 16(a), when *τ*_2_ > 14, the period and frequency of the two neurons are almost coincident, which is consistent with the phase synchronization obtained when *τ*_1_=5 in Figure 15, while when *τ*_2_ < 14, this is the same. Although the change trend is the same, the deviation is large, which means that the two neurons are asynchronous.

When *τ*_1_=10, the period and frequency (Figure 16(b)) of the two coupled neurons are almost consistent with the conclusion in Figure 14, and the coupled neurons produce certain errors in some areas. The figure shows that the period and frequency do not coincide and then start to coincide after a sharp decline, and the variation amplitude is obviously reduced, which corresponds to the phase synchronization when *τ*_1_=10 in Figure 14.

## 4. Lag Synchronization under Nonidentical Coupling

In the previous section, the influence of coupling strength and time delay on the model under identical coupling was discussed. In this section, the synchronization phenomenon of two coupled neurons under nonidentical coupling was mainly discussed. For the coupled nonidentical chaotic system, if the coupling strength is weak, the amplitudes of the coupled neurons are irrelevant, and the chaotic system will achieve phase-locking. With the increase of the coupling strength, the amplitudes will start to build a relationship, and there will be a lag synchronization phenomenon, that is, the two states are almost the same, only the time difference exists, and one system lags behind the other. At this time, the lag synchronization can be observed through the dynamic characteristics of the amplitudes. When the coupling strength exceeds a certain critical value, the system states will remain almost the same.

When the coupling strength exceeds a certain critical value, the state of the system will keep almost the same, but there will be a time delay *τ*, which makes *x*(*t*+*τ*) ≈ *y*(*t*). In order to describe the lag synchronization, a similar function is introduced as follows [51]:

(30) $$\begin{array}{c} S\left(\tau \right)=\sqrt{\frac{{\left(\bar{x\left(t+\tau \right)-y\left(t\right)}\right)}^{2}}{\sqrt{\bar{x^{2}\left(t\right)}\cdot \bar{y^{2}\left(t\right)}}}}, \end{array}$$

where $\bar{x},\bar{y}$ represents the average value, and if *S*(*τ*) is sufficiently small, it means that the two chaotic systems have achieved lag synchronization with a lag time of *τ*. In which a represents the average value, and if *S*(*τ*) is sufficiently small, it means that the two chaotic systems have achieved lag synchronization with a time delay *τ*.

**Figure 17:**
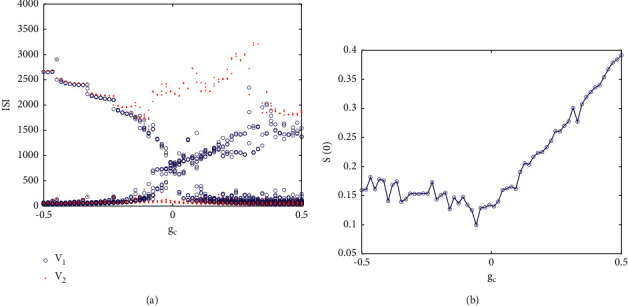
(a) The relationship between the ISI sequence of two coupled neurons and the coupling strength in the case of nonidentical and asymmetric time-delay coupling. (b) The relationship between similarity function and coupling strength.

This section mainly studies the synchronous transition of neurons under nonidentical and asymmetric time-delay coupling. First, we consider the relationship between the firing state of coupled neurons. At that time, Figure 17(a) shows the relationship between ISI and coupling strength. The blue concentric points and red solid points in the figure correspond to neurons 1 and 2, respectively. It can be observed from the figure that under negative coupling strength, the ISIs of two coupled neurons are almost the same, but with the increase of coupling strength, they show great differences, especially. In fact, under the condition of nonidentical coupling, the amplitude of two neurons is different, and it is also obvious in frequency.  Figure 17(b) shows the relationship between the similar function *S*(0) and the coupling strength. It can be clearly seen that in the positive coupling, *S*(0) monotonically increases, while in the negative coupling, the value of *S*(0) is relatively small, that is, the correlation degree of the membrane potential of two neurons is relatively low, which indicates that the two coupled neurons have achieved lag synchronization.

Second, the firing sequence of neurons with nonidentical coupling is significantly different, which is much more complex than that of a single neuron, which means that the stimulation current is an important factor affecting the firing pattern of neurons and also affects the synchronous behavior. As shown in Figures 17(a) and 17(b), the coloring diagram of *S*(0) in the parameter space (*I*_exc1_, *I*_exc2_) under different coupling strengths is irregular in both cases; the correlation degree of coupling strength *g*_*c*_=−0.5 is higher than that of *g*_*c*_=0.4. The results show that different stimulus currents are easier to achieve lag synchronization under negative coupling.

**Figure 18:**
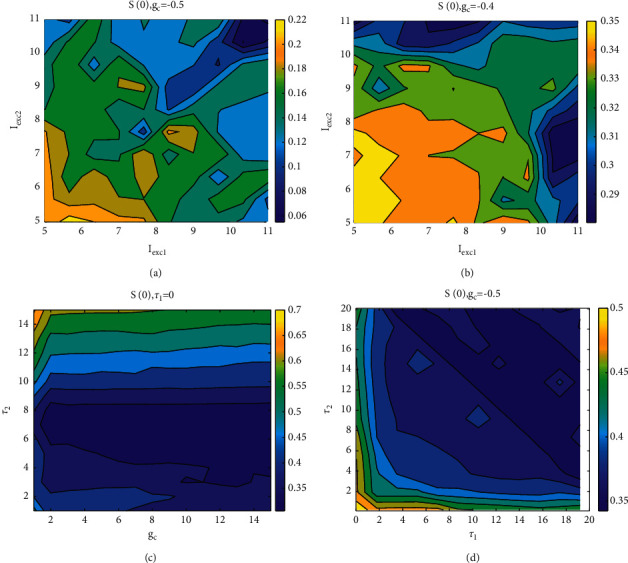
Similar functions on different two-parameter planes: (a) when *g*_*c*_=−0.5, the similar functions on the plane (*I*_exc1_, *I*_exc2_); (b) when *g*_*c*_=0.4, the similar functions on the plane (*I*_exc1_, *I*_exc2_); (c) when *τ*_1_=0, the similar functions on the plane (*g*_*c*_, *τ*_2_); and (d) when *g*_*c*_=−0.5, the similar functions on the plane (*τ*_1_, *τ*_2_).

Finally, the synchronization effect of time delays on coupled neurons is discussed, and the asymmetric time delays are discussed from two aspects. On the one hand, the similar function between time delays and coupling strength *g*_*c*_ is shown in Figure 18(c). The effect of time delays is obviously greater than that of coupling strength, and *S*(0) gradually increases with the increase of time delays, indicating that a single time delay cannot effectively increase the degree of synchronization, and the effect of negative coupling is more obvious than that under positive coupling. On the other hand, given the coupling strength *g*_*c*_=−0.5, the relationship between similarity function and asymmetric time delays is shown in Figure 18(d), which means that the lag increases at any time and the degree of synchronization is gradually enhanced, which indicates that the time delays effectively increases the lay synchronization of coupled neurons.

## 5. Conclusion

In this paper, the synchronization of two linearly coupled PBC neurons is analyzed. By calculating the ISI, correlation coefficient, maximum synchronization difference, and similarity function of the coupled chaotic system, it is found that there is a complex synchronous transition behavior in the coupled PBC network, including asynchronization, weak synchronization, and complete synchronization. The degree of synchronization between the two coupling neurons is judged by the correlation coefficient. When the correlation coefficient is 1, it can be seen that the coupling neurons are completely synchronized. When the correlation coefficient is not 1, it is incomplete synchronous. This situation contains a variety of possible weak synchronization and requires further analysis.

Several common types of synchronization are discussed based on the effect of time delay on coupled systems in this study. First, the synchronization and transition caused by coupling strength are discussed under the condition of no delay coupling. It is found that when the coupling is negative (the coupling symbol is determined by the direction of the current), the two coupling neurons undergo fully synchronous period-3 bursting, and then they get transit to approximate synchronization. With the increase of coupling strength, the coupled neurons realize the transition from asynchronous to out-of-phase synchronization. Then, symmetric time-delay coupling is discussed. Numerical simulation shows that two coupled neurons are still in complete synchronization with weak time delay. It is proved that the continuous dependence of time-delay equation on coupling strength with weak time delay combined with theory. With the increase of time delay, coupled neurons turn to an asynchronous state, and when time delay increases to a certain extent, coupled neurons begin to move repeatedly between complete synchronization and approximate synchronization (Figure 8). This synchronous transition type is more complex than the weak to the strong synchronous transition of chaotic coupled Morris–Lecar neurons [11]. In addition, on the basis of symmetric time delay, the effects of different coupling strengths are discussed, and it is found that the synchronous transition phenomenon caused by negative coupling is obviously richer than that caused by positive coupling.

Then, the asymmetric time-delay coupling is discussed, and the phase difference is defined by Poincaré mapping [13]. The results show that the firing pattern and synchronous transition of chaotic coupled PBC neurons are robust to large time delays, and the robustness is more obvious with the increase of time delays, that is, the asymmetric time delays always show the phase synchronization of chaotic firing in a large range. Robustness means that some performance indexes of the system remain unchanged under disturbance. At the beginning of the article, the influence of external stimulus current on a single neuron has been discussed and combined with bifurcation theory (Figure 3); it is proved that the bifurcation curve of periodic orbit generated from HB bifurcation is an important factor causing the increase of the peaks number. Finally, based on the previous related results, the nonidentical asymmetric time-delays coupling is discussed, and the delayed synchronization caused by stimulus current, time delays, and coupling strength is analyzed with the help of similarity function. The results show that the neurons are more likely to achieve lay synchronization under negative coupling and further show that the increase of time delays effectively improves the degree of synchronization of coupled neurons. In contrast, the role of stimulation current in synchronization is very complex, which needs further study. Generally speaking, with the increase of coupling strength, the synchronization transition process of two nonidentical coupled chaotic systems is from phase synchronization to lag synchronization and then to almost complete synchronization [51]. Although there are differences in the transfer process in this PBC coupling system, it also shows that the coupling strength can cause a complex synchronization process.

Physiological experiments have shown that neuronal synchronization is closely related to many clinical diseases [5, 6]. Therefore, it is necessary to discuss the synchronous transition of neurons. This study shows that even if it is a simple linear coupling, by controlling the coupling strength and time delay, all kinds of synchronization can be achieved effectively.

## Figures and Tables

**Table 1:** Parameter values used for the functions in the eight-dimensional model equations (4–5) and (15–17).

Parameter	Value
*C*_*m*_	21 *μ*F
*g*_Na_	28 nS
*g*_*K*_	11.2 nS
*g*_*L*_	2.3 nS
*g*_NaP_	2 n
*g*_CAN_	0.7 nS
*V*_Na_	50 mV
*V*_*K*_	−85 mV
*V*_*L*_	−58 mV
*V*_*m*_	−34 mV
*V*_*n*_	−29 mV
*V*_*p*_	−40 mV
*V*_*h*_	−48 mV
*s*_*h*_	5 mV
*s*_*m*_	−5 mV
*s*_*n*_	−4 mV
*s*_*m*_	−6 mV
$\overset{-}{\tau_{h}}$	10 ms
$\overset{-}{\tau_{n}}$	10,000 ms
*n*_CAN_	0.97
*K*_CAN_	0.74 *μ*M
*ϵ*	0.09
*d*	0.5
*Ca*_*c*_	0.1
*l*_*c*_	0.9

## Data Availability

The simulation data used to support the findings of this study are included within the article.

## Appendix

The ion current *I*_*x*_ in models (15–17) can be expressed as follows:

(A.1) $$\begin{array}{c} I_{{\text{Na}}_{i}}=g_{\text{Na}}\cdot m_{\infty }^{3}\left(V_{i}\right)\cdot \left(1-n_{i}\right)\cdot \left(V_{i}-V_{\text{Na}}\right),\\ I_{NaP_{i}}=g_{NaP}\cdot p_{\infty }\left(V_{i}\right)\cdot h_{i}\cdot \left(V_{i}-V_{\text{Na}}\right),\\ I_{{\text{CAN}}_{i}}=g_{\text{CAN}}\cdot f\left(\left[\text{Ca}\right]\right)\cdot \left(V_{i}-V_{\text{Na}}\right),\\ I_{K_{i}}=g_{K}\cdot n_{i}^{4}\left(V_{i}-V_{K}\right),\\ I_{L_{i}}=g_{L}\cdot \left(V_{i}-V_{L}\right), \end{array}$$

where *C*_*m*_ is membrane capacitance, membrane potential, and opening probability of Na^+^ channel and *K*^+^ channel, which is an index of neurons. The maximum conductance of the ion channel is reversal potential, which indicates that sodium, persistent sodium, and calcium activate nonspecific cations, potassium, and leakage, respectively.

The state function *X*_*∞*_ of gated variables is composed of the sigma function

(A.2) $$\begin{array}{c} X_{\infty }\left(V_{i}\right)=\frac{1}{\left(1+\exp\left(\left(V_{i}-V_{X}\right)/s_{X}\right)\right)},\;X\in \left\{m,p,n,h\right\}, \end{array}$$

where *V*_*X*_ is the half inactivation voltage of the gate channel and *s*_*X*_ is the corresponding slope.

(A.3) $$\begin{array}{c} \tau_{n}\left(V_{i}\right)=\frac{{\bar{\tau }}_{n}}{\text{cosh}\left(\left(V_{i}-V_{n}\right)/\left(2\cdot s_{n}\right)\right)},\\ \tau_{h}\left(V_{i}\right)=\frac{{\bar{\tau }}_{h}}{\text{cosh}\left(\left(V_{i}-V_{n}\right)/\left(2\cdot s_{n}\right)\right)}, \end{array}$$

where *τ*_*n*_(*V*_*i*_) and *τ*_*h*_(*V*_*i*_) are voltage-dependent time functions and $\bar{\tau_{n}}$ and $\bar{\tau_{h}}$ are the maximum time constants.

For convenience, make

(A.4) $$\begin{array}{c} f_{1}\left(V,n,h\right)=-\frac{\left(I_{L}+I_{K}+I_{\text{Na}}+I_{\text{NaP}}+I_{\text{CAN}}+I_{\text{exc}}\right)}{C_{m}},\\ f_{2}\left(V,n\right)=\frac{\left(n_{\infty }\left(V\right)-n\right)}{\tau_{n}\left(V\right)},\\ f_{3}\left(V,h\right)=\frac{\left(h_{\infty }\left(V\right)-h\right)}{\tau_{h}\left(V\right)}. \end{array}$$

There are many parameters in this model including Eq. (4), Eq. (5), and Eqs. (15–17), and the values used in this paper are listed in Table 1.

*f* is the hill function related to calcium concentration:

(A.5) $$\begin{array}{c} f\left(\left[\text{Ca}\right]\right)=\frac{1}{\left(1+{\left(K_{\text{CAN}}/\left[\text{Ca}\right]\right)}^{n_{\text{CAN}}}\right)}. \end{array}$$

The description of the somatic subsystem can be referred to references [29, 30], and the derivation and explanation of the imposed path about the calcium subsystem are shown in reference [49]. The numerical software in this paper is mainly XPPAUT and MATLAB, and the fourth-order Runge–Kutta algorithm is used with a step size of 0.1.
